# The Effect of Interlayer Materials on Ceramic Damage in SiC/Al Composite Structure

**DOI:** 10.3390/ma13173709

**Published:** 2020-08-21

**Authors:** Jiawei Bao, Yangwei Wang, Rui An, Bowen Zhang, Huanwu Cheng, Fuchi Wang

**Affiliations:** 1 School of Materials Science and Engineering, Beijing Institute of Technology, Beijing 100081, China; baojiawei993@163.com (J.B.); 3120195536@bit.edu.cn (R.A.); zhangbw@bit.edu.cn (B.Z.); chenghuanwu@bit.edu.cn (H.C.); wangfuchi@bit.edu.cn (F.W.); 2 National Key Laboratory of Science and Technology on Materials under Shock and Impact, Beijing 100081, China

**Keywords:** ceramic damage, interlayer, fiber-reinforced resin composites, impact, numerical simulation

## Abstract

The effect of interlayer materials on the damage of ceramics in the SiC/Al composite structure was analyzed through experiments and simulation. Using 0.25 mm thermoplastic polyurethane (TPU) as a reference, a 0.5 mm aramid fabric (AFRP) or a 0.5 mm carbon fiber reinforced epoxy matrix composite (CFRP) was added to the interlayer, respectively. Through the impact tests, it was discovered that the ceramic damaged area in the TPU composite structure was severe. With the addition of AFRP, the damaged area of the ceramic layer was reduced by 73% under the same impact conditions. The addition of CFRP also reduced the damage of ceramics. The evolution process of the tensile stress on the ceramic rear surface was presented by simulation. The tensile evolution process analysis can explain the experimental phenomenon well and can be used to predict the damage of the ceramics.

## 1. Introduction

In the field of armor protection, the ceramic/metal, ceramic/fiber laminate are the classic layered composite structures, which are usually bonded by adhesives [1,2,3]. The existence of the intermediate layer will affect the stress wave transfer process, and then modify the damage pattern of the ceramic layer and the backing plate.

The existence of the adhesive layer can generally improve the ballistic property of ceramic/backing plate structures. Previous reports [4,5] showed that the potent combination of the intermediate layer and ceramic layer could significantly improve the energy absorption capacity of the structure by increasing the energy dissipated at the interface. A strong bonding between ceramic and backing plate can also benefit for forming a complete ceramic cone by prolonging the dwell time. Surface treatment methods were effective in enhancing the bonding strength. For instance, Harris [6] increased the bonding ability between ceramics and resins by refired process or laser treatment. The research found that an increased stiffness in the adhesive layer could reduce the cumulative damage of ceramic layers in multi-hit experiments. Jang [7] introduced nanowire and nanohole on the surface of aluminum alloy and alumina. An increasing surface area and roughness of adhered surfaces resulted in the smallest damage of the ceramic and the smallest bumps on the backing plate in the study.

When the thickness and type of the intermediate layer change, it will affect the ballistic resistance of the ceramic and the overall structure. The result of Rashed [8] and Kong [9] showed that increasing the thickness of the intermediate layer will reduce the ballistic resistance of the ceramic/metal structure. TPU and epoxy are two typical representative adhesives of thermoplastic, and thermoset adhesives, respectively. Researches [9,10,11] presented that the ceramic tiles bonded by TPU did not fall off after the impact, while the ceramic tiles bonded by epoxy all fell off. However, after the penetration of the projectile, the structure bonded with epoxy has a more significant effect on the crushing of the projectile since the epoxy has better support for ceramic than TPU. The researchers also reported that the sound velocity of the adhesive affected the deformation area of the backing plate. The larger the sound velocity, the larger the deformation area of the backing plate.

In addition to using the adhesive as an intermediate layer, researchers also added other materials to the intermediate layer and obtained many impressive results. Wang [12] placed silica gel, WC, epoxy between ceramic matrix composites and homogeneous armor steel. Using tests by split Hopkinson pressure bar (SHPB), it was found that almost all incident waves were reflected when silica gel was used. When using WC, due to its sizeable acoustic impedance, a strong compression wave was observed in the ceramic material composites, and this compression wave can improve the ballistic resistance. Tasdemirc [13] also investigated the effect of the intermediate layer on wave propagation by SHPB. When rubber was laterally constrained, the transmitted wave increased to 200 MPa, which was 60 MPa when there was no restraint. The study pointed out that Teflon can delay plastic wave propagation to the backing plate and reduce the strength of the wave. This process can maximize the performance of the ceramic and avoid extensive damage to the backing plate [14]. Foam materials are also used as an intermediate layer besides adhesive materials. It was found that closed-cell foam aluminum between the ceramic layer and fiber-reinforced resin laminates can make the broken area more concentrated and the ceramic debris finer. The foam material was equivalent to a "wave filter" for stress wave transmission. Wave strength transmitted to the backing plate was significantly reduced and led to reducing the back bulge [14,15]. Wang [16] and Liu [17,18] explained the relationship between the intermediate layer and the backing layer. The intermediate layer needed to have sufficient fracture toughness and high bending strength to support ceramics. The primary role of the backing layer was not to resist the projectile directly but to control the deformation of the intermediate layer.

It can be seen that the intermediate layer will affect the ballistic resistance of the whole structure. Previous studies mostly used low-impedance materials like adhesives and foams, focusing on the transmission of waves or damages to the backing layer. Little attention was paid to the detriment of the ceramic layer. However, in practical applications, the damage of ceramics is closely related to the multi-hits resistance ability of the armors. Moreover, there was little literature on the use of fiber composites for the intermediate layer. In this study, the fiber composite materials were introduced into the intermediate layer. The effect of the intermediate layer on ceramic damage was investigated through impact tests and numerical simulation. This study was expected to reduce the damaged area of ceramics without significantly increasing the weight of the structure.

## 2. Test Procedure and Method

### 2.1. Materials

Hexagonal silicon carbide ceramic (SiC) tiles were provided by China North Materials Science and Engineering Technology Group Corporation (Jinan, Shandong Province, China). The specifications and typical indexes are as follows: a length of 30 mm, a thickness of 5 mm, a hardness of HRA 92 ± 1, a bending strength of 370 ± 20 MPa, and a fracture toughness of about 4 MPa·m^−1/2^. Aramid plain weave fabric (AF) was provided by the Chengrand Research Institute of Chemical Industry Co. Ltd. (Chengdu, Sichuan Province, China). Fiber fineness was 3000D, and the thickness of a single layer was 0.5 mm. China Aerospace Composite Center provided CFRP material. The matrix resin was epoxy, the resin mass fraction was 40%, and the single-layer thickness was 0.25 mm. TPU films were provided by TianJin Xin Bao Glass Co., Ltd. (Tianjin, China). TPU films existed in the form of a thin film with specifications of 0.6 mm and 0.3 mm. The aluminum alloy was a 2024-T351 aluminum alloy with a thickness of 5 mm.

### 2.2. Preparation of Structures

**Figure 1:**
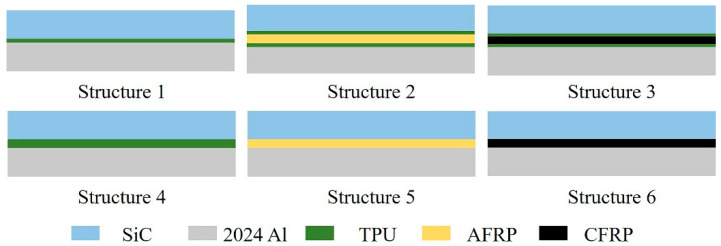
Structures diagram.

**Figure 2:**
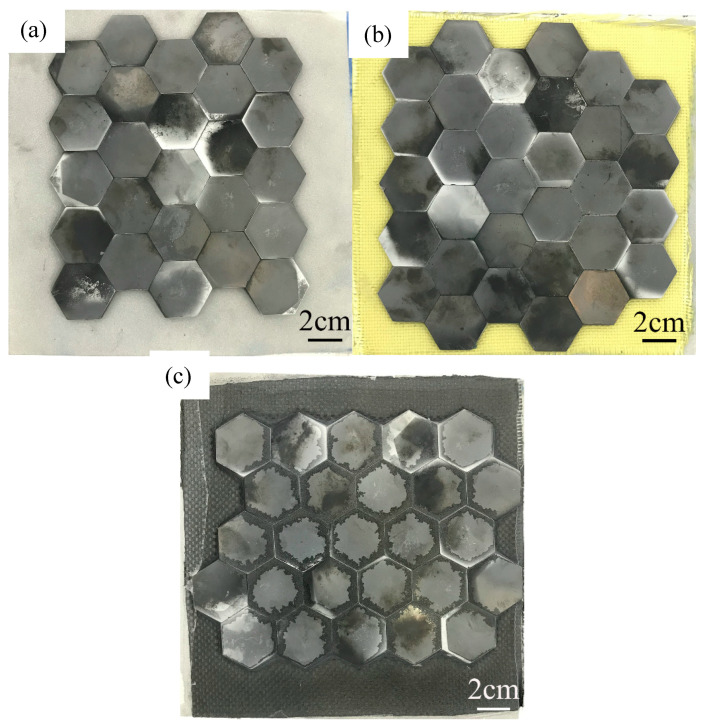
Front view of the three structures: (**a**) Structure 1; (**b**) Structure 2; (**c**) Structure 3.

The ceramic/intermediate layer/aluminum alloy structures were prepared by an autoclave method. Table 1 shows the structures in detail and Figure 1 presents the schematic diagrams. The bonding surface of aluminum alloy was sandblasted to remove grease and dirt on the surface for improving the mechanical occlusion strength between the aluminum alloy and the intermediate layer. The fabrication process was: the heating rate was 3 ℃/min, and the temperature was kept at 120 ℃ for 2 h. The vacuum degree in the bag was minus 0.098 MPa, and the external pressure was 1.2 MPa. The pressure was released after the temperature was lower than 60 ℃. Figure 2 presents the front view of the ceramic layers.

### 2.3. Impact Tests

**Figure 3:**
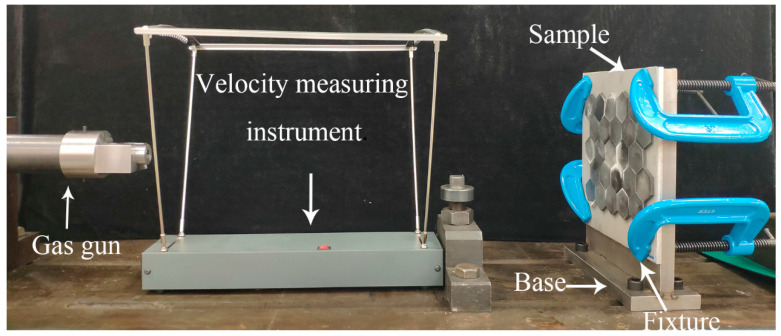
Impact test devices.

The test device is shown in Figure 3. The device consisted of a gas gun, projectiles, velocity measuring equipment, base, and fixtures. The projectiles were spherical projectiles with a diameter of 10 mm. Bearing steel with the hardness HRC 62–66 was selected as the projectile’s material. The compressed gas was used to accelerate the projectile. The distance between the muzzle and the target plates was 60 cm. By controlling the pressure of the gas, projectile velocity was controlled from 150 m/s to 180 m/s.

### 2.4. Simulation Method

Based on the LS-DYNA software, the process of the impacts on different structures was simulated in the study. Due to the high hardness of the projectile, the armor-piercing (AP) projectile’s material was used [19]. JH-2 model was used for SiC ceramic materials. According to the performance of the SiC ceramics used in the experiment, the parameters referred to previous studies [20,21,22,23]. Table 2 presents the parameters of SiC ceramic. The material model of TPU was mat finite elastic strain plasticity. The parameters of rubber were used given that its characteristics are similar to rubber [24,25,26]. Table 3 lists the parameters of TPU. Aramid fabric interacted with TPU to form aramid/TPU composites (AFRP) during the auto-clave process. Therefore, the parameter was based on Kevlar/thermoplastic resin composites [27,28]. The setting for CFRP was referred to carbon fiber/epoxy composites [26].

The model of AFRP and CFRP was mat composite damage, and the parameters were shown in Table 4. The total dimension of the plate in the simulation was 200 mm × 200 mm. For the projectile impact area, the element size was 0.5 mm × 0.5 mm × 0.5 mm. For the area away from the impact area, the element size was 1 mm × 1 mm × 1 mm. Table 1 shows the correspondence between the simulated structures and the actual structures.

The contact between the projectile and the target plates was eroding surface to surface, and the contact between the layers in structures was automatic surface to surface tiebreak. Normal and shear failure stresses for the ceramic-TPU interface were 25 MPa, and 10 MPa, respectively. For the TPU-AFRP interface, these two parameters were as same as the ceramic-TPU interface. For the TPU-CFRP interface, normal and shear failure stresses were 60 MPa, and 25 MPa, respectively. 

## 3. Results and Discussion

### 3.1. Impact Tests’ Results

Table 5 depicts the increase in the number of damaged ceramic tiles with an increasing number of shots. Since the impact energy was small, the aluminum alloy had not undergone plastic deformation and no damage occurred on the backing plates. The most impact energy of the projectiles was consumed by ceramics. 

In addition to the larger damaged area caused by the first shot of Structure 1, the damaged area of subsequent shots was 2–3 tiles on average. Specifically, the damaged area of Structure 2 was the smallest in that it was 73% smaller than that of structure 1. Although, Structure 3 had the largest average damage area per shot, the damaged degree was still lower than Structure 1—this will be discussed in the following section.

**Figure 4:**
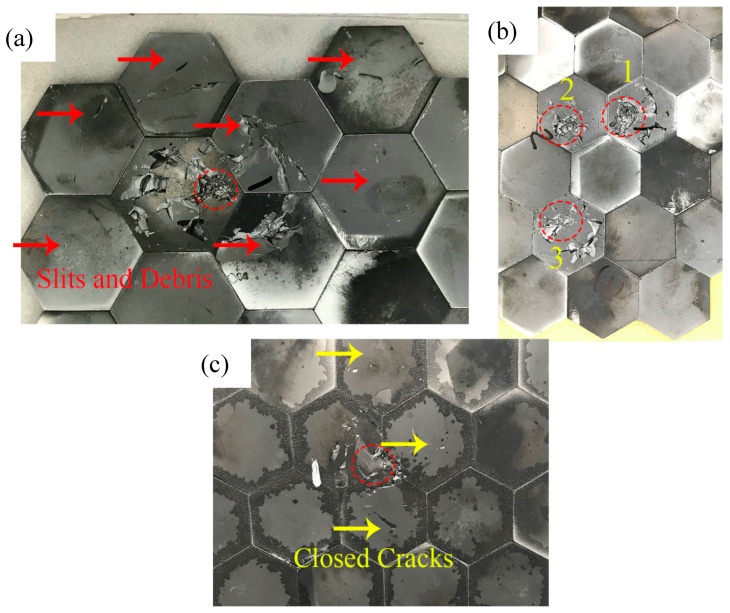
The first impact damage of the three structures: (**a**) Structure 1; (**b**) Structure 2; (**c**) Structure 3.

Figure 4 indicates the first damage morphology of the three structures. The red dotted line marked the impact location, and the yellow arrows point to the cracks. For the structure 1, the impact location of the first projectile was located on a single piece of ceramic. All seven ceramic tiles around the impact point were observed to have visible cracks, and some ceramic debris fell from the surfaces after impact. On the contrary, although the first shot’s impact location in Structure 2 was similar to the first shot in Structure 1, there were no excessive cracks in adjacent ceramics. This phenomenon attributed to the increased thickness of the intermediate layer, which alleviated the stress caused by metal elastic deformation. The addition of AFRP also affected the transmission process of the stress wave. The transmission process of the stress wave will be discussed in the following simulation part.

For Structure 2, even after the third shot, the ceramic tiles around the three impact points had no visible cracks, reflecting excellent ceramic damage reduction ability. When another fiber-reinforced resin material—CFRP, which had higher modulus, higher strength but lower deformation ability compared with AFRP, was added in the intermediate layer. The first impact point was located at the junction of the three ceramics. Although four cracks were generated, the cracks were all closed and small, and no debris fell from the surface, compared with structure 1, the phenomenon of ceramic debris falling off was not noticeable, and the structure also demonstrated superior ceramic damage reduction ability.

**Figure 5:**
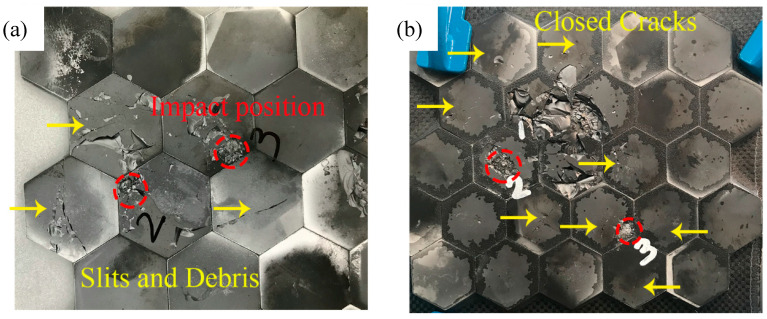
Ceramic damage morphology after the third penetration: (**a**) Structure 1; (**b**) Structure 3.

Figure 5 shows the ceramic damage morphology after the third impact. The impact positions of Structure 1 and Structure 3 were similar. However, the damage morphology was different. For Structure 1, the ceramic damage was serious. Large cracks with slits and debris showed on the surface of the ceramic tiles. The ballistic properties of these ceramic tiles had been reduced a lot and can be judged to be invalid when facing the next impact. For Structure 3, the third projectile impact point in structure 3 was still located at the junction of the three ceramics. Notably, there were only visible closed cracks and no debris around the impact zone. That meant these ceramic tiles still can resist penetration by the next projectile.

**Figure 6:**
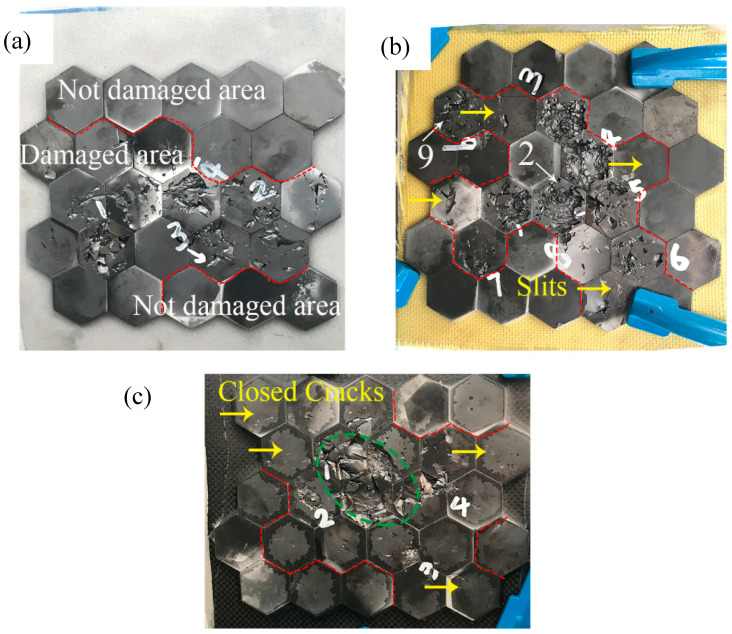
Final ceramic damage morphology of the three structures: (**a**) Structure 1; (**b**) Structure 2; (**c**) Structure 3.

Figure 6 shows the final ceramic damage morphology of Structure 1 to Structure 3. With the TPU layer, Structure 1 was impacted by four projectiles and the damage of the ceramic gradually expanded under the impact of the subsequent impacts. Specifically, the undamaged area accounted for 10/25, approximately 40%. Compared with Structure 2 and Structure 3, the damage characteristic of structure 1 was that the damaged area was extensive but the impacted ceramic tiles were still kept on the structure. No single tile completely damaged and fell off like Structure 2 (shot 1 to shot 4) and Structure 3 (shot 1). It illustrated that the existence of the TPU layer spread the damage to the surrounding areas and reduced the damage of the impacted tiles. 

For Structure 2, Structure 2 was impacted by a total of nine projectiles because of its excellent ceramic damage reduction ability. Although the damaged area was limited to a single tile of ceramic, the damage of the single tile was very serious. Ceramics, at the first to the fourth impact points have been completely felled, leaving only the bottom of the ceramic cone glued to the TPU. In particular, at the second impact position, a complete ceramic cone has been formed at the bottom of the ceramic and the radial cracks were obvious. The presence of ceramic cone suggested that the energy consumption characteristic of ceramics was fully exploited [5,31,32]. Besides the four ceramic tiles marked by yellow arrows, the other ceramic tiles that were not penetrated showed no visible cracks. After the nine projectiles penetrated, the undamaged area accounted for 14/31, approximately 45%. From the above phenomenon, it can be concluded that the presence of AFRP made the damaged area more concentrated.

Structure 3 was impacted by four projectiles, the ceramic damage at the first impact location became more serious (the green dotted line area) after the subsequent impacts. There were only seven tiles of undamaged ceramics, accounting for 7/26, approximately 27%. Considering the fact that Structure 3 had two projectiles impacted on the junction of three ceramics, and the cracks were closed (yellow arrows) with no slits. Therefore, the effect of restricting ceramic damage in Structure 3 was also considered significant. Comparing Structure 1 to Structure 3 comprehensively, the damage degree of the impacted ceramic tiles sorted from serious damage to minor damage: Structure 2 >Structure 3 > structure 1. But in terms of the damage degree of the not impacted ceramic tiles, the order from serious to light was as follows: Structure 1 >Structure 3 >Structure 2.

### 3.2. Simulation Results of Impact Tests

For a better understanding of the impact process, the simulation was conducted to examine the influence of the intermediate layer on the stress propagation of ceramics. Notably, the hydrostatic pressure of the units on ceramic rear surfaces at a range of 0 mm–50 mm from the impact position in the ceramic part was extracted. 

**Figure 7:**
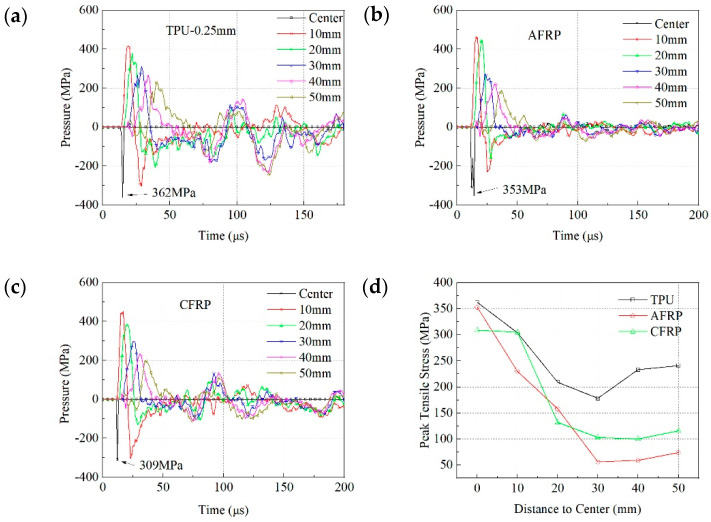
Hydrostatic pressure-time curve of the units when the different intermediate layer was used: (**a**) TPU-0.25 mm; (**b**) AFRP; (**c**) CFRP; (**d**) relationship between peak tensile stress and position.

Figure 7 indicates the hydrostatic pressure versus the time curve of these units under different intermediate layers. The compressive stress is positive, and the tensile stress is negative in the definition of hydrostatic pressure. Since the tensile stress wave reflected to the ceramic through the ceramic/intermediate layer interface was the main reason for forming initial cracks in ceramics, the change of the tensile stress with time represented the damage progress of the ceramics. When TPU was used as the intermediate layer, the maximum tensile stress at the impact point was 362 MPa. The tensile stress value at the unit that was 10 mm away from the impact point was close to 300 MPa. As the distance increased and penetration became more severe, the peak tensile stress at different locations was still maintained at a relatively large value. This was especially true of the units that were 40 mm and 50 mm away from the impact point where the tensile stress was still 244 MPa around 125 µs. On this basis, it can be concluded that the surrounding ceramics suffered severe damage.

For structure 2, the peak tensile stress at the impact point was 353 MPa, which was slightly lower than the value of the TPU structure. However, the maximum tensile stress of the unit at 10 mm reduced to 250 MPa at 25 µs, which was 23% lower than that of the TPU structure. Additionally, the tensile stresses of the other units were lower than 70 MPa after 50 µs. This showed that with the addition of AFRP, the intensity of reflected tensile stress around the impact point, especially at positions beyond 30 mm, has been greatly reduced. The corresponding result in the experiments was aligned well with the simulation results.

When CFRP was incorporated into the intermediate layer, the peak tensile stresses at the impact point and the 10 mm unit were almost 309 MPa. The tensile stress of the other units periodically fluctuated in the subsequent response, and the peak value of the tensile stress was about 100 MPa, which was 43% higher than the AFRP structure but 59% lower than the TPU structure. This specifically states that, while the damaged area was equivalent to that of the TPU structures, the damage degree of the ceramic was between the TPU structure and the AFRP structure.

**Figure 8:**
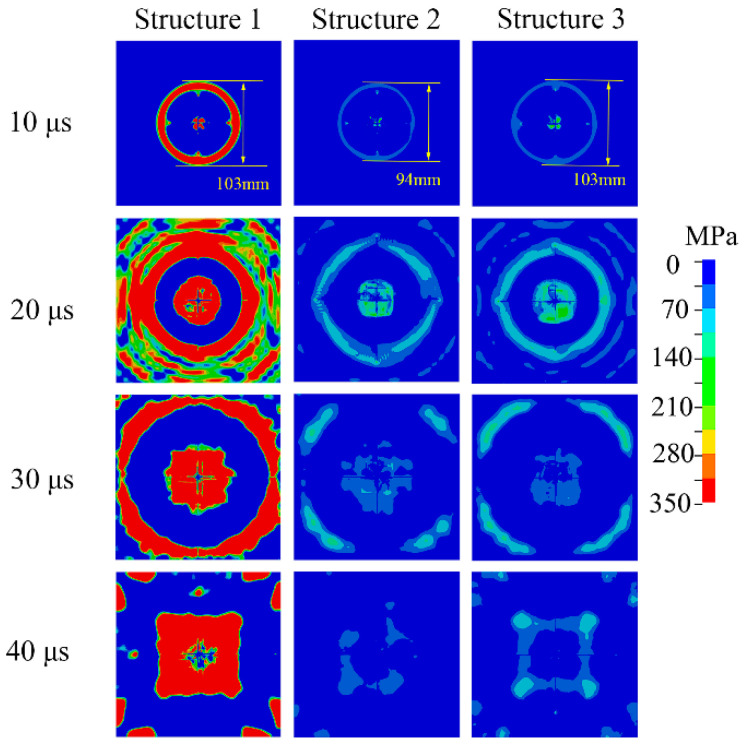
The evolution of the tensile stress of the ceramic’ rear surface with time.

Figure 8 depicts an evolutionary cloud diagram of the tensile stress on the ceramic’s rear surface till 40 μs. For structure 1, a circle with large tensile stress appeared at a diameter of approximately 50 mm. With time, the region with nearly 350 MPa tensile stress expanded outwards. At 40 μs, there was still a tensile stress circle with 310–350 MPa. After adding AFRP, the tensile stress was a low value at 10 μs. Both the magnitude of tensile stress and the area of the stress circle were the smallest of the three structures. For Structure 3, the damaged area was almost the same as that of structure 1. Nonetheless, the tensile stress amplitude was larger than that of Structure 2, but did not exceed 140 MPa. This proved that the surrounding ceramics only suffer mild damage.

From the above observation, it can be concluded that the simulation results aligned well with the results of the experiment. Through analysis of tensile stress evolution on the ceramic’s rear surface, ceramic damage can be predicted.

### 3.3. Further Simulation and Discussion

From the perspective of the wave transmission process, if the wave transmission process in the structure is simplified to a one-dimensional stress wave process, the stress analysis is similar to SHPB tests. Table 6 depicts the acoustic impedance values of different materials as well as the reflection and transmission coefficients to SiC ceramics. Elastic wave impedance, the reflection and transmission coefficients can be calculated by:

(1) $$A=\rho \cdot C=\rho \cdot \sqrt{\frac{E}{\rho }}=\sqrt{E\rho }$$

(2) $$R=\frac{A_{\mathrm{inter}}-A_{\mathrm{SiC}}}{A_{\mathrm{inter}}+A_{\mathrm{SiC}}}$$

(3) $$T=\frac{2A_{\mathrm{inter}}}{A_{\mathrm{inter}}+A_{\mathrm{SiC}}}$$

The meaning of each letter is as follows:

A: acoustic impedance value.

Suffixes: different materials.

ρ: the density of the material.

C: elastic wave velocity.

E: elastic modulus

R: reflection coefficients

T: transmission coefficients

Since AFRP and CFRP were anisotropic material, the acoustic impedance values of AFRP and CFRP were calculated by simulation parameters as no available test values can be used. The acoustic impedance of the TPU is the lowest apparently. Consequently, the reflection coefficient to the SiC ceramic is the largest, which indicates that the stress amplitude of the reflected tensile wave is also the largest. On the contrary, the CFRP has a relatively larger acoustic impedance than the TPU. Under such circumstances, the reflected stress amplitude of the tensile wave is the lowest of the three materials. The calculation results are consistent with the simulation results. It should be noted that this is only a simple calculation of one-dimensional stress wave and that the actual three-dimensional stress wave transmission process is more complicated.

TPU, AFRP and CFRP represent three types of typical materials:

- Incompressible material with significant deformation capacity, low modulus and low strength;
- Materials with certain deformability, high in-plane strength, relatively low modulus and rigidity in the thickness direction;
- Materials with brittleness, high in-plane strength, large modulus and density in the thickness direction.

The above experiments and simulations were based on the addition of AFAP or CFRP to 0.25 mm TPU, respectively. The thickness of the entire intermediate layer was different. Considering the difference caused by the thickness, the thickness of the intermediate layer was fixed to 0.5 mm. Through the same simulation parameters and analysis methods, the differences caused by the properties of three different materials were investigated. The intermediate layers of the three structures were 0.5 mm TPU, 0.5 mm AFRP (no TPU), and 0.5 mm CFRP (no TPU).

**Figure 9:**
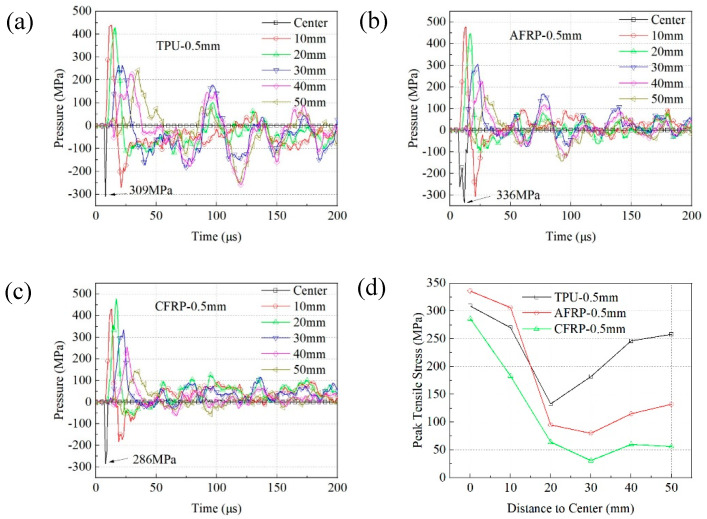
Curve of hydrostatic pressure in the ceramic rear surface versus time: (**a**) TPU; (**b**) Kevlar Composites; (**c**) CFRP; (**d**) relationship between peak tensile stress and position.

Figure 9 demonstrates the hydrostatic pressure versus time curve of units at different positions on the rear surfaces of the ceramics. When the thickness of TPU increased to 0.5 mm, the peak tensile stress at the impact point was 309 MPa, which was 14.6% lower than 362 MPa for Structure 1. However, the maximum tensile stresses at the other positions, especially those that were 40 mm and 50 mm from the impact point, were still higher than 250 MPa. It showed that the increase in the thickness of the TPU layer did not alleviate the problem that the tensile stress around the impact point was still exceeded 250 MPa.

For Structure 6, the peak tensile stress at each position was lower than that of structure 5. This was especially true for the units at 30 mm, 40 mm and 50 mm away from the center, where the stress can be reduced by 60% of structure 5’s value. Moreover, the maximum tensile stress at the 20 mm position was already reduced to 64 MPa. This indicated that high-impedance CFRP could reduce the tensile stress of the ceramics compared with TPU and AFRP.

Compared with the presence of 0.15 mm TPU on the upper and lower surfaces, when only CFRP or AFRP was used as the intermediate layer, the peak tensile stress at the center was reduced by 5–7%. This suggested that the addition of low-impedance material TPU will increase the tensile stress on ceramics. 

Based on the above experimental results and simulations, the most ideal characteristics that the intermediate layer material should have are large acoustic impedances and strong deformability. The increase of acoustic impedances is beneficial in reducing the stress amplitude of the reflected tensile wave. Under such circumstances, the deformability of the material is very important. Large deformability of the material can allow it to follow the movement of the ceramic under the instantaneous impact, and restore it to its original states after penetration, which helps to achieve reliable adhesion.

## 4. Conclusions

Impact test results have demonstrated that the intermediate layer material has a significant impact on ceramic damage. In the case of solely using a 0.25 mm TPU layer, a large area of ceramics was shattered, which was caused by low impedance characteristics of the TPU. After adding a 0.5 mm AFRP or a 0.5 mm CFRP to the intermediate layer, the damage of the single ceramic tile and destruction area of the ceramic layer will be reduced under the same penetration condition. Among them, the combination of TPU + AFRP + TPU has the best damage reducing ability. Compared with the 0.25 mm TPU layer, it can reduce the damaged area of ceramics by 73%.

Through the analysis of the tensile stress evolutionary process in simulation, the experiments’ results were well explained. Based on the simulation, the damage of ceramics can be predicted and further controlled by changing intermediate layers. Simulation results of the same thickness of the three materials reflected that the ceramic damage was the smallest when only 0.5 mm CFRP was used. Overall, by adding the intermediate layer, the destination of reducing ceramic damage without significantly increasing the weight of the structure was achieved.

## Figures and Tables

**Table 1:** Structures’ illustration.

Number	Intermediate Layer Thickness in Experiments (mm)	Intermediate Layer in Simulation
Structure 1	0.26	0.25 mm TPU
Structure 2	0.75	0.15 mm TPU + 0.5 mm AFRP + 0.15 mm TPU
Structure 3	0.80	0.15 mm TPU+ 0.5 mm CFRP + 0.15 mm TPU
Structure 4	-	0.5 mm TPU
Structure 5	-	0.5 mm AFRP
Structure 6	-	0.5 mm CFRP

**Table 2:** JH-2 parameters for SiC.

Parameters	Value
Density, ρ(kg/m^3^)	3200
Shear modulus, G (GPa)	193
Intact normalized strength parameter, A	0.96
Fracture normalized strength parameter, B	0.35
Strength parameter for strain rate dependence, C	0
Fractured normalized strength parameter, M	1
Intact strength parameter, N	0.65
Maximum tensile strength, MPa	370
Maximum normalized fractured strength, Sfmax	0.8
Hugoniot elastic limit, HEL (GPa)	13
Pressure component at the Hugoniot elastic limit, PHEL (GPa)	5.9
Beta	1.0
Parameter for plastic strain to fracture, D1, D2	0.48, 0.48
Pressure coefficients, K1, K2, K3	204, 0, 0

**Table 3:** Parameters for TPU.

Density, ρ (kg/m^3^)	Elastic Modulus, E (MPa)	Poisson’s Ratio, ν	Yield Stress, SIGY (MPa)	Tangent Modulus, ETAN (MPa)	Failure Strain
1100	25	0.495	10	80	2.5

**Table 4:** Parameters for AFRP and CFRP [27,28,29,30].

Parameters	AFRP	CFRP
Density, ρ(g·cm^−3^)	1.18	1.59
Elastic modulus, Ex (GPa)	14.63	63.90
Elastic modulus, Ey (GPa)	14.63	62.70
Elastic modulus, Ez (GPa)	4.30	8.19
Shear modulus, Gxy (GPa)	6.98	3.44
Shear modulus, Gxz (GPa)	6.98	3.27
Shear modulus, Gyz (GPa)	6.98	3.25
Poisson’s ratio, ν_xy_	0.048	0.048
Poisson’s ratio,ν_zx_	0.18	0.051
Poisson’s ratio,ν_zy_	0.18	0.051
In-plane tensile strength, Xt (MPa)	365	769
In-plane tensile strength, Yt (MPa)	365	823
In-plane compressive strength, Yc (MPa)	113	916
In-plane shear strength, Sc (MPa)	67	77
Normal tensile stress, Sn (MPa)	62.8	60.0
Tranverse shear strength, Ss (MPa)	22.9	50.0
Erosion Effective Strain	1.5	0.1

**Table 5:** Increased number of damaged ceramic tiles in impact tests.

Shot Number	Structure 1	Velocities in Structure 1 (m/s)	Structure 2	Velocities in Structure 2 (m/s)	Structure 3	Velocities in Structure 3 (m/s)
**1**	8	161.3	1	166.6	4	168.8
**2**	3	170.4	1	162.4	5	162.3
**3**	2	171.9	1	176.2	4	167.9
**4**	2	166.6	1	164.6	6	170.1
**Sum of 4**	15	-	4	-	19	-
**5–9**	-	-	13	163.5, 169.7, 169.7, 164.0, 159.5	-	-
**Total**	15	-	17		19	-

**Table 6:** Acoustic impedances of intermediate materials.

Material	Density g/cm^3^	Modulus in the Thickness Direction (GPa)	Acoustic Impedance in the Thickness Direction (g·cm^−2^·s^−1^×10^5^)	Sound Velocity in-Plane Direction (m/s)	Reflection Coefficient	Transmission Coefficient
SiC	3.18	441	25.5 [33]	12020 [34]	0	1
TPU	1.15	-	1.80	1700	−0.87	0.13
AFRP	1.23	3.62	2.11	-	−0.85	0.15
CFRP	1.59	5.89	3.06	-	−0.79	0.21
